# VITAMIN D RECEPTOR GENE MUTATIONS AND VITAMIN D SERUM LEVELS IN ASTHMATIC CHILDREN

**DOI:** 10.1590/1984-0462/;2018;36;3;00016

**Published:** 2018

**Authors:** Hevertton Luiz Bozzo Silva Santos, Silvia de Souza e Silva, Estela de Paula, Lilian Pereira-Ferrari, Liya Mikami, Carlos Antônio Riedi, Herberto José Chong-Neto, Nelson Augusto Rosário

**Affiliations:** a Universidade Federal do Paraná, Curitiba, PR, Brasil.; b Faculdades Integradas do Brasil, Curitiba, PR, Brasil.; c Centro Universitário Autônomo do Brasil, Curitiba, PR, Brasil.

**Keywords:** Asthma, Vitamin D, Genetic polymorphism, CDX2 transcription factor, Pediatrics, Asma, Vitamina D, Polimorfismo genético, Fator de transcrição CDX2, Pediatria

## Abstract

**Objective:**

To verify the relationship between polymorphisms of the vitamin D receptor gene (VDR), clinical findings, and serum vitamin D (VD) levels in asthmatics.

**Methods:**

A cross sectional study of 77 children aged 7 to 14 years old, who were attended at a specialized clinic. The children were divided into 3 groups: asthmatics who had been using inhaled corticosteroids (ICS) for more than one year; asthmatics who had not been using ICS; non-asthmatics, and children without allergies (according to the International Study of Asthma and Allergies in Childhood ­- ISAAC). Spirometry, skin prick tests, the presence of a VDR promoter CDX2 polymorphism from an allele-specific polimerase chain reaction (PCR), exons 2 and 3 polymorphisms genotyping by PCR-SSCA (*single-strand conformational analysis*), total immunoglobulin E (IgE) and specific IgE to mites and grass were evaluated in these three groups. Levels of 25-hydroxyvitamin D were determined in asthmatics only.

**Results:**

The mean age of the children was 10.8±2.0 years old, 57% were male, 38 were asthmatic and using ICS, 22 were asthmatic and not using ICS, and 17 were non-asthmatic. Allergic rhinitis was present in 90% of asthmatics. Homozygous CDX2 was detected in 23% of the patients and absent in the control group (p=0.03). Lower forced expiratory volume in 1 second (FEV_1_%) values were observed in CDX2 homozygous asthmatics (p=0.001). Variations in the exon 2 and 3 sequences were not related to asthma or the other tests. VD deficiency or insufficiency was detected in 98% of asthmatics. There was no association between VD levels and genetic polymorphisms from exons 2 and 3.

**Conclusions:**

There was a positive association between homozygous CDX2 polymorphism, asthma and lower FEV_1_% values. CDX2 is capable of modifying cell interaction between VDR and VD, and it could be associated with the prevalence of asthma, and the difficulty in controlling the disease.

## RESUMO

**Objetivo:**

Verificar a relação dos polimorfismos do gene do receptor de vitamina D (RVD) com sinais clínicos e níveis de vitamina D (VD) em asmáticos.

**Métodos:**

Estudo transversal com 77 crianças de 7 a 14 anos de um ambulatório especializado, divididas em 3 grupos: asmáticos, em uso de corticoide inalatório (ICS) por mais de um ano; asmáticos sem necessidade de ICS; não asmáticos e não alérgicos (de acordo com o *International Study of Asthma and Allergies in Childhood* - ISAAC. Foram avaliados: espirometria, testes alérgicos, presença do polimorfismo CDX2 do promotor do RVD por reação em cadeia da polimerase (PCR) e genotipagem de polimorfismos dos éxons 2 e 3 por PCR-SSCA (*single-strand conformational analysis*), imunoglobulina E (IgE) total e IgE específica para ácaros e gramíneas nos três grupos estudados. Níveis de 25-hidroxivitamina D foram dosados nos asmáticos.

**Resultados:**

A média de idade foi 10,8±2,2 anos, 57% masculinos, 38 asmáticos com ICS, 22 sem ICS e 17 não asmáticos. Rinite alérgica esteve presente em 90% dos asmáticos, polimorfismo CDX2 em 23% dos asmáticos e ausente nos controles (p=0,03). Menores níveis de volume expiratório forçado no primeiro segundo (VEF_1_%) foram observados nos asmáticos homozigotos para CDX2 (p=0,001). Variações nas sequências dos éxons 2 e 3 não foram relacionadas com a asma ou demais testes. Deficiência ou insuficiência de VD foi diagnosticada em 98% dos asmáticos. Não houve associação entre níveis de VD e polimorfismos genéticos dos éxons 2 e 3.

**Conclusões:**

Observou-se associação positiva entre polimorfismo CDX2 em homozigoze com asma e menores valores de VEF_1_%. O CDX2 pode modificar a interação celular do RVD com a vitamina, bem como pode estar associado com a asma e com a dificuldade de controle da doença.

## INTRODUCTION

Asthma is the most common childhood chronic illness.^1^ It arises from the interaction between genetic factors, exposure to the environment, and other specific factors that lead to the development and continuation of symptoms.

Vitamin D (VD) deficiency is related to a higher incidence of asthma and other allergic diseases.^2^ ^,^ ^3^ ^,^ ^4^ Dietary intake accounts for a small part of the daily requirement of VD. As populations become more prosperous and westernized, people spend more time indoors, have less exposure to sunlight, and use sunscreen. As such, the prevalence of hypovitaminosis D increases.

Perinatal VD deficiency is capable of affecting lung and fetal immune system development, and may be exacerbated by post-natal VD deficiency.^5^ Low VD levels were associated with an increased frequency of asthma,^6^ particularly in boys,^7^ exacerbated asthma symptoms and more severe seizures,^2^ a greater severity of atopic dermatitis^8^ and a higher frequency of anaphylaxis.^9^ However, the exact relationship between VD and allergic diseases remains unknown. Although VD supplements may reduce the risk of seizures in asthma patients,^10^ it is not currently possible to recommend the use of VD in the prevention and treatment of allergic diseases.^11^ ^,^ ^12^

In order for VD to properly function, its receptor must be produced and work correctly. The vitamin D receptor (VDR) is a nuclear protein, which is composed of 437 amino acids, encoded by the VDR gene located on chromosome 12. The VDR is composed of 11 exons and spans 75 kb.^13^ More than 900 single nucleotide polypmorphisms (SNP) were identified in this gene, including coding and non-coding regions. Most of these are concentrated in exons 2 and 3, which are responsible for encoding the DNA binding domain. Alterations in these two exons modify the zinc finger that binds to DNA, generating a deformation at the receptor, which prevents the vitamin from binding.^14^ Changes in the 5’ region of the VDR gene promoter can alter expression patterns and messenger ribonucleic acid (mRNA) levels, whereas variations in the 3’ region (UTR) may affect mRNA stability and the efficiency of protein translation.^15^ The CDX2 polymorphism is derived from the substitution of an adenine (A) with a guanine (G), in a 1e portion of the VDR gene promoter region at position 4913, altering VDR transcription levels and the overall activity of nuclear receptor transcription, which is responsible for modulating the genes that affect inflammation, immunoregulation, and the remodeling of airways.^16^

Knock-out mice for VDR have high levels of interleukin (IL) 13 and immunoglobulin E (IgE).^17^ Mutations in the VDR gene have been associated with colorectal cancer, precocious puberty, atopic dermatitis, and other allergic diseases in certain populations.^18^ Changes in this gene modify its mechanisms, thus preventing or hindering the activity of the VD, even in individuals with normal levels of the vitamin.

The objectives of this study were: to identify the frequency of the CDX2 polymorphisms of the promoter region and the polymorphisms of the exons 2 and 3 of the VDR gene in a sample of asthmatic patients, as well as to determine if there is any relationship between these polymorphisms and the diagnosis of asthma, VD and calcium serum levels, and the need for corticosteroids.

## METHOD

The study had an observational, analytical and cross-sectional design, in which individuals aged 7 to 14 years old participated. A convenience sample was recruited on the day of the consultation in a specialized outpatient clinic. Two groups were identified: individuals diagnosed with persistent asthma according to the Global Initiative for Asthma (GINA 2010), using inhaled corticosteroid (ICS) ≥ 400 mcg / day of beclomethasone or an equivalent for a period of more than 1 year, and individuals with intermittent asthma, who did not use ICS. A third group with the following characteristics were included in order to serve as a comparison group for the presence of VDR gene polymorphisms in non-asthmatics: patients with no history of asthma and/or atopy, patients considered to be non-asthmatic and non-atopic, and patients that age-matched the asthmatics. These patients were randomly selected according to when they arrived to collect exams requested by other outpatient clinics of the Hospital de Clínicas of the Universidade de Paraná (HC-UFPR). We excluded individuals with a clinical diagnosis of associated chronic diseases, as well as non-asthmatic patients who answered affirmatively to a question on the International Study of Asthma and Allergies in Childhood (ISAAC) questionnaire.

Clinical data on asthmatics were obtained from standardized medical charts. Spirometry [percent predicted forced expiratory volume in the first second (% FEV_1_)] was obtained using a Koko^®^ Spirometer (NSPIRE Health, Longmont, CO, USA), with reference spirometric values from Polgar and Promadhat^19^ and allergic skin tests using a skin prick for *Dermatophagoides pteronyssinus* (DP), *Blomia tropicalis* (BT) and *Lolium perene* (LP) with IPI ASAC extracts from Brazil (São Paulo, Brazil) were registered.

Levels of total and specific IgE for DP, BT, and LP were obtained from all of the individuals. For statistical purposes, total IgE values above 5,000 international units per mL (IU/mL) were considered in ascending order as 5.001, 5.002, 5.003 etc. IU/ mL; and specific IgE values above 100 KU / L, in the same manner, were considered in ascending order for 101, 102, 103 etc. KU / L.

Levels of 25 hydroxyvitamin D [25 (OH) VD], morning cortisol, parathyroid hormone (PTH), calcium, phosphorus and alkaline phosphatase levels were determined in asthmatics. The reference values for VD were: levels <30 ng / mL = insufficiency; levels <15 ng / mL = deficiency; levels ≥30 ng / mL = normal.^20^ ^,^ ^21^

To evaluate the promoter region of the VDR gene, we used the allele-specific polymerase chain reaction (PCR) with the following primers: A-for 5’TCCTGAGTAAACTAGGTCACAA3’, A-rev 5’ACGTTAAGTTCAGAAAGATTAATTC3’, G-for 5’AGGATAGAGAAAATAATAGAAAACATT3’ e G-rev 5’AACCCATAATAAGAAATAAGTTTTTAC3’.

Reactions were performed at a final volume of 20 mcL and contained 18 mcL of PCR supermix (Invitrogen™, Carlsbad, CA, USA), 150 ng DNA, 16.4 pM of primer A-for, 20.3 pM of primer A-ver, 18.8 pM of primer G-for, and 18.7 pM of primer G-ver. The PCRs were performed in a thermal cycler and the amplification cycles were the same for each pair of primers, using the following programming:

1. 94ºC for 5 minutes;
2. 94ºC for 45 seconds;
3. 56ºC for 45 seconds;
4. 72ºC for 45 seconds; repeat steps 2 to 4 28 times;
5. 72ºC for 5 minutes.

The set of primers G-for and G-rev specifically amplify the G allele, generating a 110 bp fragment. A-for and A-rev specifically amplify the A allele that is 235 bp in size. Primers G-for and A-rev amplify the internal control fragment that is 297 bp in size.^22^ The fragments were separated by size in 2.5% agarose gel electrophoresis at 125 V for 1 h. Gel staining was then performed with ethidium bromide and was revealed in an ultraviolet transilluminator.

For the evaluation of polymorphisms in exons 2 and 3, a PCR-SSCA (single-strand conformational analysis) was used. For exon 2, the primers used were a 2a sense (5’AGCTGGCCCTGGCACTGACTCTGCTCT3’) and a 2a antisense (5’ATGGAAACACCTTGCTTCTTCTCCCTC3’) and for exon 3, 3a sense primers (5’AGGGTGAAGGAGCCGGAAGTTCAGTGAC3’) and 3a antisense primers (5’CTTTCCCTGACTCCACTTCAGGCCCAA3’) were used.

The PCR reaction for each exon was 20 mcL, and contained 18 mcL PCR Supermix (Invitrogen), 150 ng DNA and 18 pM of each primer. The PCR reaction for exon 2 was performed by 35 touchdown cycles starting with an annealing temperature at 60 ° C and ending at 50 °C. For exon 3, 35 amplification cycles were used, as described: 94°C for 1 minute, 48°C for 1 minute, and 72°C for 1 minute.

The samples, after the amplification reaction, generated, for exon 2, a fragment of 267 bp and, for exon 3, a fragment of 220 bp. After PCR, the amplified product was subjected to 10% polyacrylamide gel electrophoresis, and the run was performed at 250 V for 2 hours and 30 minutes. Gel staining was performed with silver nitrate.^23^

Clinical characteristics, frequency of VDR gene polymorphisms according to the presence of asthma, and a diagnosis of asthma according to genotype, were presented in frequency distribution and proportions, and for comparison, Fisher’s exact test was used. Dosages of specific and total IgE were presented in medians and, to make comparison between groups, the Kruskal Wallis test was used. The data were stored in Microsoft Excel^®^ (MicrosoftTM Redmond, USA) and were processed with Action^®^ software (Estatcamp^®^, São Carlos, Brazil). Univariate relationships between serum levels of 25 (OH) VD and patient demographic characteristics were determined using the Pearson correlation coefficient, with p values being ≤0.05.

The study was approved by the Ethics Committee on Human Research of the HC-UFPR, and a free and informed consent form was signed by all of the parents.

## RESULTS

The genotypes of 77 individuals were evaluated: 38 asthmatics using ICS; 22 asthmatics not using ICS; and 17 non-allergic individuals. Forty-four (57%) patients were males and their mean age was 10.8 ± 2.2 years. The mean body mass index (BMI) among the boys was 18.2 ± 3.0 kg/m^2^ and, among the girls, it was 18.6 ± 3.5 kg/m^2^.

When assessed individually, the asthmatics group using ICS, the asthmatics not using ICS, and non-asthmatics group were similar with regard to personal and family history of atopy. Pulmonary function tests were performed on 50 asthmatics. There were no differences in clinical characteristics among the asthmatics (Table 1).

**Table 1:** Clinical characteristics of the asthmatic group.

	No ICS (n=22) n (%)	ICS (n=38) n (%)	p-value
Rhinitis	20 (90.9)	37 (97.4)	0.54
Conjunctivitis	15 (68.2)	22 (57.9)	0.58
Atopic dermatitis	3 (13.6)	5 (13.2)	1.00
Other allergies	2 (9.1)	2 (5.3)	0.61
Family history of asthma	16 (72.7)	27 (71.1)	1.00
History of smoking	9 (40.9)	11 (28.9)	0.40
Hospitalizations	4 (18.2)	17 (44.7)	0.05
AST	22 (100)	36 (94.7)	0.52
OPD	3/19 (15.8)	7/31 (22.6)	0.72
PBD +	4/19 (21.1)	11/31 (35.5)	0.35
FEV_1_% (median)	102	98	0.87
Years with the disease (average±SD)	9.1±2.2	8.7±2.5	0.54

ICS: inhaled corticosteroid use; AST: allergy skin test; OPD: obstructive pulmonary disease; PBD: Post bronchodilator test; FEV_1_%: forced expiratory volume in the first second; SD: standard deviation.

Almost 98% of the individuals tested had inadequate levels of VD, and there was no difference between groups of asthmatics that were or were not using ICS.

Total and specific IgE levels for PD, BT, and LP were higher in the asthmatic groups using and not using corticosteroids, when compared to the control group (p <0.001). The total and specific IgE median values were verified from the distribution of the patients in the three groups, according to the genotype in the promoter region (Table 2).

**Table 2:** Medium immunoglobulin E (kU/L) values according to the genotype of the promoter region of the gene.

IgE	GG	GA	AA	p-value*
DP	44.7	33.2	101.0	0.01
BT	16.5	3.2	27.3	0.08
LP	0.3	0.1	0.6	0.06
Total	775.0	498.0	1108.5	0.06

IgE: immunoglobulin E serum total (kU/L); GG: homozygotes for the allele G; GA: heterozygotes for G and A; AA: homozygotes for allele A. DP: *Dermatophagoides pteronyssinus*; BT: *Blomia tropicalis*; LP: *Lolium perenne*; *Kruskal-Wallis test.

There was no significant correlation between serum levels of VD and other laboratory tests performed on 60 asthmatics. There was also no significant correlation between serum levels of VD, values of FEV_1_, and the number of positive allergens obtained from the allergic skin test (47 and 60, respectively, of asthmatics). The Pearson correlation test was applied to variables with normal distribution (calcium, phosphorus, cortisol, parathormone, alkaline phosphatase, specific IgE for PD, BT, LP and total IgE) and did not show any significance.

For an analysis of the VDR gene, three genotypes from the promoter region were evaluated in the 77 individuals. CDX2 polymorphism was present in 71.4% of the population evaluated (71.6% of asthmatics and 70.5% of non-asthmatics). The data point to a trend (p = 0.06) of an association between genotype of the promoter region and the diagnosis of asthma (Table 3). Also in Table 3, the presence of exon 3 mutations in 7 of the 69 individuals tested was observed. There was no association between the presence of the mutation and the diagnosis of asthma. No individuals were identified with genetic variations in exon 2.

**Table 3:** Frequency of the polymorphism of the vitamin D receptor genes in accordance with the presence of asthma.

Region	Genotype	Asthma		Control		p-value*
		n	%	n	%	
Promoter	GG	17/60	28.3	5/17	29.4	0.06
	GA	29/60	48.3	12/17	70.6	
	AA	14/60	23.3	0	0	
Exon 2	Usual	51/51	100	14/14	100	
Exon 3	Variant	7/52	13.5	0	0	0.18
	Usual	45/52	86.5	17/17	100	

GG: homozygotes for the G allele; GA: heterozygotes for the G and A alleles; AA: homozygotes for the A allele; *Fisher’s exact test.

In order to better evaluate the association between the presence of the CDX2 polymorphism and a diagnosis of asthma, the subjects were compared as follows: homozygous group for the A allele (AA) versus the Gx group [homozygous for the G allele (GG) allele and heterozygotes (GA)]. Subsequently, the GG group versus the Ax group, which is composed of AA and GA, was compared. From this analysis, 46 asthmatics and 17 controls had the Gx genotype, while 14 asthmatics and no controls had the AA genotype. An analysis of the contingency table shows an association between the homozygous for the A allele and the diagnosis of asthma (p = 0.03) (Table 4). The evaluation of individuals with Ax versus GG genotypes showed no association with the diagnosis of asthma (p = 1.00). There was also no association between inhaled corticosteroid therapy and the polymorphisms being studied.

**Table 4:** Number of individuals diagnosed with asthma according to genotype.

Genotype	Asthma (n=60) n (%)	Controls (n=17) n (%)	p-value*
AA	14 (23.3)	0 (0)	0.03
Gx	46 (76.6)	17 (100)	

AA: homozygote for the A allele; Gx: homozygotes for the G allele (GG) and heterozygotes (GA); *Fisher’s exact test.

Asthmatics were divided into 2 groups (usual genotype and variant genotype) according to the presence of mutations presented for exon 3. The promoter region was divided into three groups, according to the genotype (GG, GA and AA). There was no significant difference between groups for VD, calcium, phosphorus, PTH, cortisol, and number of positive allergic tests. A significant difference (p = 0.004) was observed for the mean percentage of FEV_1_ predictors among asthmatics when grouped according to promoter region genotypes (GG: 98.2%, GA: 102.5% and AA: 79.5%). When comparing the FEV_1_% of the Gx (100.8%) versus the AA (79.4%) groups, a significant difference was observed (p = 0.001). This relation was not observed in the Ax (96.0%) versus GG (98.2%) analysis (p = 0.71).

## DISCUSSION

The occurrence of hypovitaminosis D has been widely reported, both among healthy individuals and those with allergic diseases.^24^ A possible explanation for the low serum levels of VD found in the studies may be due to the inadequacy of the reference values used.^20^ Initially, the reference values were based on health individuals, who had little sun exposure. This method is not very accurate, given that VD levels are related to sun exposure, diet, latitude of residence, skin color, lifestyle and age.^4^ Since VD levels are dependent on multiple variables, there is a need to establish reference values ​​for each population. Nevertheless, the serum level of VD that is associated with the incidence of diseases was established to be 30 ng / dL.^20^ ^,^ ^21^

In the evaluated sample, the promoter polymorphism, 4913G> A, known as CDX2, was frequent in the tested population (71.4%) and was positively associated with the diagnosis of asthma, when it was homozygous. In the heterozygotes, for any mutation, including the CDX2 mutation, the expression of two different proteins can occur. However, the structural changes of the variant protein on phenotype expression (which are sufficient to cause clinical consequences) determine the pattern of dominance.^25^ In this case, similar to heterozygotes, where 50% of the protein is normally expressed, it can be inferred that, in asthma, the G allele has a dominant pattern, because it is associated with the lower prevalence of the disease.

In addition to being associated with a higher prevalence of asthma, the presence of the A allele also demonstrated a relationship with lower values of FEV_1_ observed among asthmatics. Although worse pulmonary function values ​​were associated with a greater severity of the disease, the presence of the CDX2 mutation in this group did not result in a greater severity of the disease, as it was similarly distributed among both asthmatics treated with inhaled corticosteroids (persistent asthma) and asthmatics that did not require treatment (intermittent asthma).

Genetic variations of exon 3 were uncommon in the study population and did not influence the diagnosis, treatment or laboratory parameters.

Although it is associated with the diagnosis of asthma, the CDX2 polymorphism has been shown to have a protective effect in other studies. It was related to the promoter’s higher transcription rate and, consequently, to a possible increase in the intestinal absorption of calcium.^26^ It has also been associated with a lower risk of fractures, osteoporosis and increased bone density.^27^

Other polymorphisms have been investigated and are, in fact, associated with an increased frequency of asthma and other allergic diseases in population studies. However, the influence of these polymorphisms on how the VDR and other genes function remains unclear.^28^ It is not yet clear how these mutations relate to asthma and other allergies.

Deficiency or insufficiency of VD was verified in 98% of the evaluated individuals. There was no relationship between VD levels with any of the polymorphisms studied. Similarly, there was no relationship between VD values and the use of ICS. There was no correlation between VD and FEV_1_ values, which is in disagreement with the literature, which showed an inverse association between VD levels and FEV_1_% values.^29^

Lower VD serum levels were related to a higher frequency of allergic diseases or asthma. Although less stringent reference values, which considered values ​​greater than 20 ng / dL, were used as artifice in this group, ^30^ more than three-quarters of the evaluated subjects did not reach adequate levels of the vitamin.

Low levels of cortisol serum were found in 17 (20%) asthmatic patients. However, this frequency was similar among those treated with ICS. Thus, it is not possible to state that such levels are due to the use of the drug. It is probable that the use of nasal topical corticosteroids to control rhinitis (present in approximately 90% of this group) is related to this result. Another hypothesis is that corticoids could have been prescribed to treat acute illnesses or crises in other centers and, therefore, was not recorded in the medical record, and may have been omitted, forgotten or even be unknown to the interviewees.

Although the results were not presented, changes in calcium, phosphorus, alkaline phosphatase and PTH levels were uncommon and they occurred in a similar fashion among asthmatics, with no statistical significance.

Serum Ige levels are inversely associated with serum VD levels.^31^ However, there is no evidence of a linear relationship between serum IgE levels and 25(OH)VD.^32^ When there are VD levels, sensitivity to home allergens like dust mites is common in asthmatic children.^6^ Natural exposure to allergens leads to increased inflammatory reactions and increased bronchial responsiveness, as well as to elevated eosinophil counts in the bronchoalveolar lavage, just like hypovitaminosis D.^29^ Although VD is correlated to asthma in a manner similar to IgE, there is a lack of experimental studies that demonstrate how this interaction actually occurs.

This study provided information about the association between CDX2 polymorphism, and the VD receptor gene promoter region, with the diagnosis of asthma, and lower FEV_1_ values. However, there are some limitations to consider, including the use of a convenience sample and the possible undeclared use of corticosteroids in the group of asthmatics supposedly not using ICS to control the disease. Another limitation exists with regard to the comparison group, which was selected to serve only as a control for the mutations. Therefore, it is suggested that an intervention study with asthmatics be performed in order to demonstrate if there is a decrease in the need for corticosteroids to control the disease after the use of VD supplements.

Since hypovitaminosis D has been linked to an increased frequency of asthma and other allergic diseases, the vitamin D receptor may be involved in the mechanism of the disease. Similarly to CDX2, it is possible that other polymorphisms or mutations in the VD receptor gene, which are capable of modifying cellular interaction with the vitamin, are associated with the prevalence of asthma and, perhaps, with the difficulty of controlling the disease.

It was concluded that the presence of CDX2 polymorphism was frequent (71.4%). The unspecified polymorphisms of exons 2 and 3 occurred discretely (7 and 2%, respectively). CDX2 polymorphism was associated with the diagnosis of asthma, as well as with lower FEV_1_% results in spirometry. There was no correlation between VD levels and the polymorphisms studied, nor was there a correlation between serum calcium levels and these polymorphisms. There was no significant association between inhaled corticosteroid therapy for the control of asthma and the polymorphisms studied.
